# de Winter electrocardiogram pattern evolving into Wellens electrocardiogram pattern in post-percutaneous coronary intervention therapy: a case report

**DOI:** 10.1093/ehjcr/ytaf139

**Published:** 2025-04-01

**Authors:** Xianghong Ma, Mengwei Bao

**Affiliations:** Department of Cardiology, the Second Hospital of Tianjin Medical University, Tianjin Institute of Cardiology, Tianjin Key Laboratory of Ionic-Molecular Function of Cardiovascular Disease, Tianjin 300211, China; Department of Cardiology, the Second Hospital of Tianjin Medical University, Tianjin Institute of Cardiology, Tianjin Key Laboratory of Ionic-Molecular Function of Cardiovascular Disease, Tianjin 300211, China

**Keywords:** Case report, de Winter syndrome, Wellens syndrome, Acute coronary syndromes, Percutaneous coronary intervention

## Abstract

**Background:**

The de Winter electrocardiogram (ECG) pattern and Wellens ECG pattern are rare but critical ECG findings, often considered high-risk equivalents of ST-segment elevation myocardial infarction (STEMI) associated with proximal left anterior descending coronary artery (LAD) occlusion, necessitating urgent angiography and reperfusion. Limited literature documents cases where the de Winter ECG pattern transitions into the Wellens pattern following percutaneous coronary intervention (PCI).

**Case summary:**

A 73-year-old man with a history of intermittent chest pain over 1 year presented with exacerbation lasting 3 h, leading to hospitalization for acute coronary syndrome (ACS). During chest pain, the ECG displayed the de Winter ECG pattern, prompting immediate emergency coronary angiography. Angiography revealed 90% stenosis at the proximal LAD, subsequently treated with drug-eluting stent placement. The stent expanded well, and LAD forward blood flow was at thrombolysis in myocardial infarction Level 3. Within 24 h post-PCI, the ECG evolved into Type B Wellens pattern, accompanied by the resolution of chest pain. The patient was discharged on the seventh day post-admission.

**Discussion:**

The de Winter ECG pattern signifies transient severe coronary stenosis during episodes of chest pain. In contrast, the Wellens ECG pattern typically occurs before PCI, with its T-wave changes often appearing during pain-free intervals, indicating spontaneous coronary artery revascularization. Post-PCI, Wellens ECG changes may suggest reperfusion injury. Timely and repeated ECG monitoring in ACS patients is crucial for identifying high-risk ECG patterns and initiating urgent reperfusion therapy. However, a Wellens ECG pattern in pre-cordial leads does not invariably signify LAD occlusion or severe stenosis.

Learning points

- de Winter electrocardiogram (ECG) pattern is a transient state and occur during episodes of chest pain, indicating acute severe proximal left anterior descending coronary artery (LAD) stenosis. Electrocardiogram should be done timely and repeatedly for acute coronary syndrome (ACS) patient to identify high-risk ECG patterns and start urgent reperfusion therapy.
- Wellens ECG pattern may occur after percutaneous coronary intervention, the ECG changes explainable by reperfusion injury. Wellens ECG changes are non-specific, not always indicate acute severe proximal LAD stenosis.

## Introduction

The de Winter electrocardiogram (ECG) pattern and Wellens ECG pattern are rare but critical ECG findings, often considered high-risk equivalents of ST-segment elevation myocardial infarction (STEMI) associated with proximal left anterior descending coronary artery (LAD) occlusion, necessitating urgent angiography and reperfusion.^1,2^ Limited literature documents cases where the de Winter ECG pattern transitions into the Wellens pattern following percutaneous coronary intervention (PCI).

## Summary figure

**Figure:**
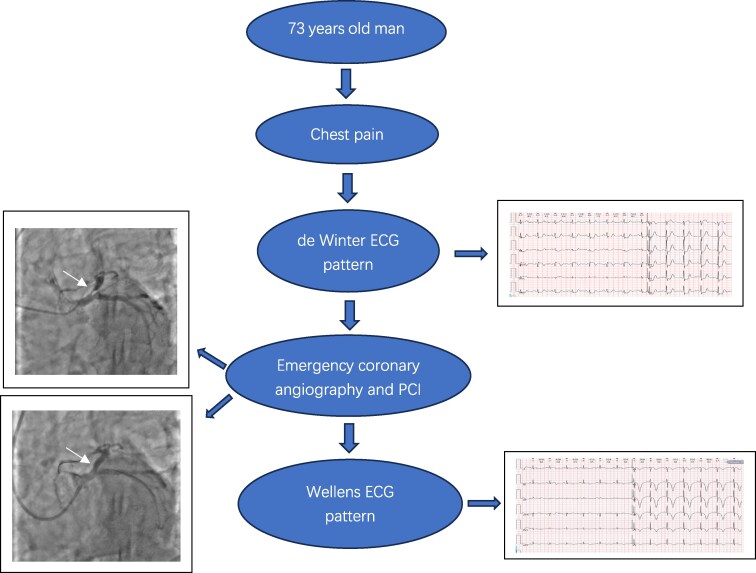


## Case summary

**Figure 1:**
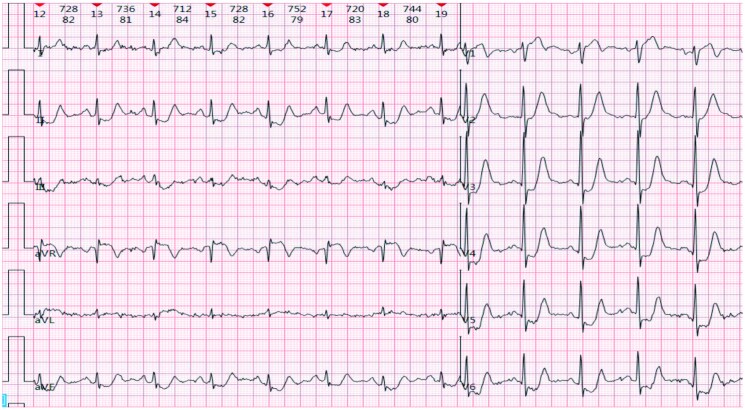
During persistent episodes of chest pain, the electrocardiogram revealed ST-segment depression in Leads II, III, and aVF, upsloping ST-segment depression at the J-point and tall, symmetric T waves in Leads V2 through V6. In addition, ST-segment elevation in Lead aVR was observed, consistent with the de Winter pattern.

**Figure 2:**
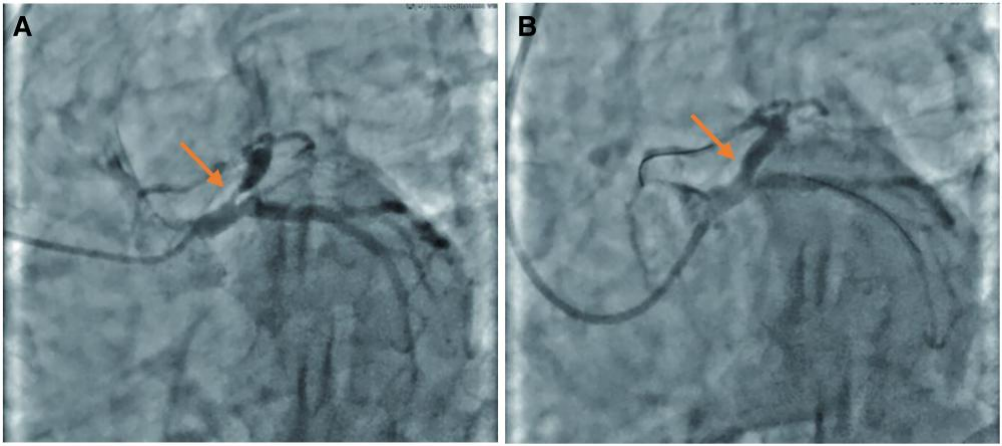
Coronary angiography and percutaneous coronary intervention. (*A*) Coronary angiography showed 90% stenosis at the proximal left anterior descending coronary artery. (*B*) A drug-eluting stent was implanted in the left anterior descending coronary artery.

**Figure 3:**
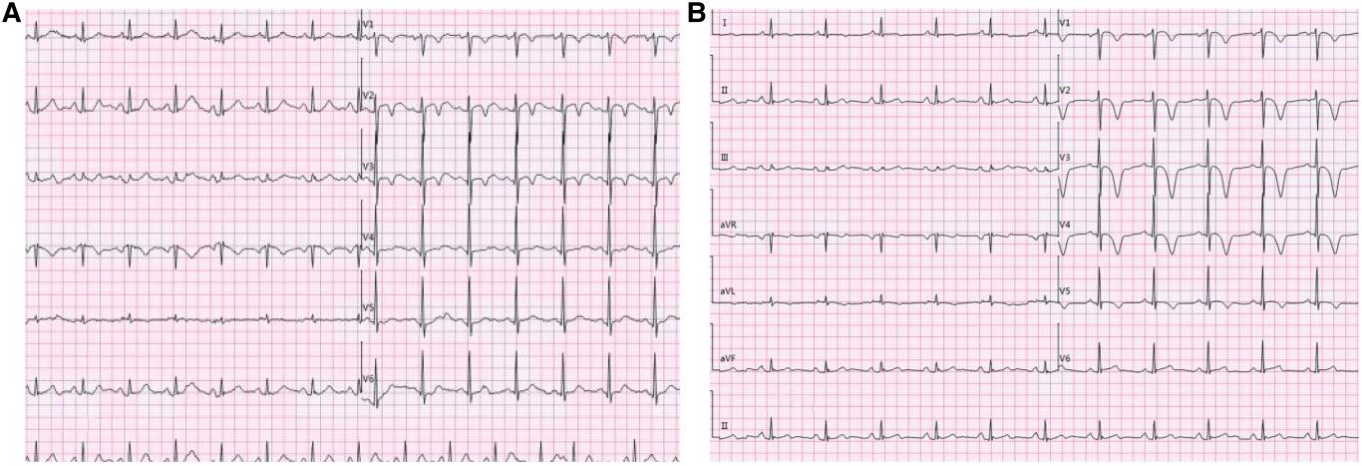
(*A*) The patient returned to the ward post-operation without reporting any specific discomfort; the electrocardiogram indicated resolution of anterior lead ST-segment depression and T-wave inversion in Leads V1–V3. (*B*) Fifteen hours post-percutaneous coronary intervention, the electrocardiogram showed T-wave inversion in Leads V1–V5, deeper than before.

**Figure 4:**
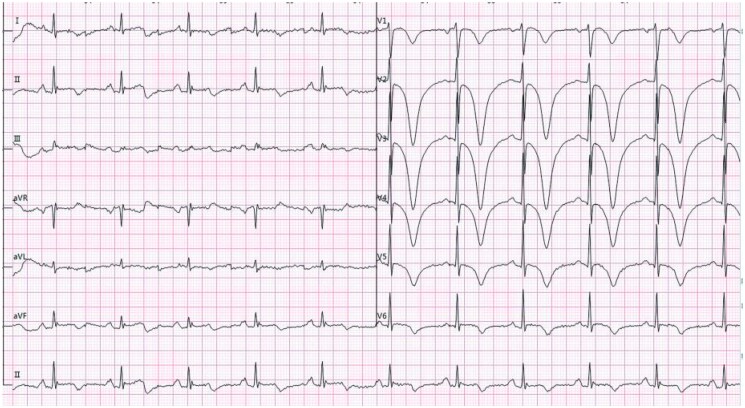
Twenty-four hours post-percutaneous coronary intervention, the electrocardiogram displayed ST-segment depression in Leads V1 through V6, and symmetric deep inversion of T-wave, consistent with Wellens pattern (Type I).

**Figure 5:**
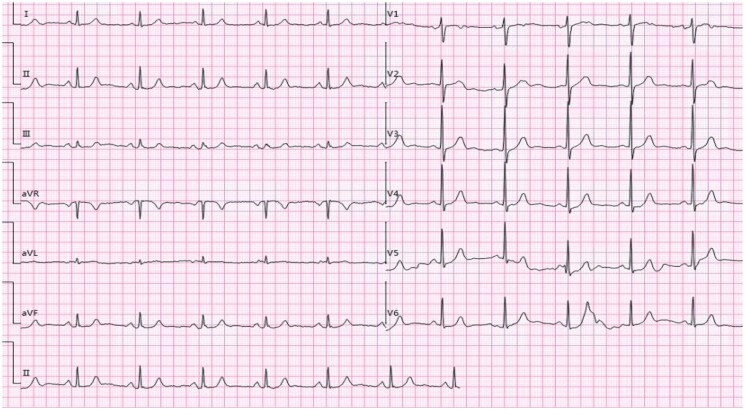
Two months post-discharged, the electrocardiogram showed no significant abnormalities.

A 73-year-old man experienced intermittent chest pain for over 1 year, with a recent exacerbation lasting 3 h. More than a year before admission, the patient had sporadic chest discomfort without apparent triggers. Upon taking nitroglycerin, the symptoms would temporarily subside after a few minutes, leading to intermittent episodes that were often disregarded. Three hours before admission, the symptoms intensified significantly, with increased frequency and prolonged duration, unrelieved by sub-lingual nitroglycerin. The patient was admitted for acute coronary syndrome (ACS) and has a history of hypertension for 5 years; there was no history of diabetes, dislipidaemia, smoking, or a family history of premature atherosclerotic cardiovascular disease. Physical examination revealed blood pressure of 132/86 mmHg, clear mental status, absence of lung rales, heart rate of 78 b.p.m., regular heart rhythm without murmurs, soft abdomen, and no lower extremity oedema. Laboratory findings showed troponin I at 0.1058 ng/mL (normal range: 0–0.0268 ng/mL), N-terminal pro-B-type natriuretic peptide at 191.5 ng/L (normal range: 0–125 ng/L), total cholesterol at 4.08 mmol/L (3.36–5.70 mmol/L), triglycerides at 0.81 mmol/L (normal range: 0.38–1.61 mmol/L), high-density lipoprotein at 1.56 mmol/L(normal range:1.07–1.90 mmol/L), low-density lipoprotein at 2.27 mmol/L(1.50–3.36 mmol/L), creatine kinase (CK) at 367.2 U/L(0–190 U/L), and CK-myocardial band at 45.7 U/L(0–24 U/L). Echocardiography revealed left atrial enlargement, segmental left ventricular motion, normal left ventricular systolic and diastolic function, and left ventricular ejection fraction of 59%. The patient was admitted with a diagnosis of ACS. After admission ECG showed no ST-T changes, the patient continued to experience intermittent chest pain, which progressively worsened and prolonged. Electrocardiogram performed during the patient’s chest pain is shown as de Winter ECG pattern (see *Figure 1*). Immediate emergency coronary angiography was performed. Following administration of 300 mg aspirin and 300 mg clopidogrel, the patient was transferred to the catheterization laboratory. Coronary angiography revealed 90% stenosis of proximal LAD with thrombolysis in myocardial infarction (TIMI) flow Grade 3 (*Figure 2A*) 50% proximal stenosis of the circumflex artery with TIMI flow Level 3, and no stenosis in the right coronary artery with TIMI flow Level 3. A drug-eluting stent was implanted in the proximal LAD post-angiography (*Figure 2B*). The stent expanded well, with no residual stenosis and dissection, and LAD forward blood flow was at TIMI Level 3. Post-procedure, the patient returned to the ward with alleviation of chest pain and no specific discomfort. Immediate follow-up ECG (*Figure 3*) showed resolution of anterior lead ST-segment depression, returning to equipotential line, T-wave inversion in Leads V1–V3. Fifteen hours post-PCI, follow-up ECG (*Figure 4A*) showed symmetric deep T-wave inversion in anterior leads. Twenty-four hours post-PCI, another ECG (*Figure 4B*) showed symmetric deep T-wave inversion and prolonged QT interval, consistent with Wellens syndrome ECG changes (Type B). However, the patient remained asymptomatic. Post-PCI, the patient continued on medical therapy. Due to timely vessel intervention, there were no post-PCI complications, and the patient was discharged on the seventh day of hospitalization. Two months after discharge, the ECG showed ST segments returning to the equipotential line and upright T waves in Leads V1 through V6 (*Figure 5*).

## Discussion

Acute coronary syndrome present with sudden onset and rapid changes, delayed diagnosis, and treatment can lead to worse prognoses. Electrocardiogram is crucial for identifying ACS and should be performed promptly and repeatedly. Recognizing characteristic ECG changes associated with acute coronary artery occlusion guides decisions for immediate reperfusion therapy.

The de Winter syndrome, first reported by de Winter *et al.*^1^ in 2008, describes a novel ECG pattern indicating proximal LAD occlusion. Instead of typical ST-segment elevation, this syndrome manifests as 1–3-mm upsloping ST-segment depression at the J-point in Leads V1–V6, followed by tall, positive, symmetrical T waves. Additionally, there may be 1–2-mm ST-elevation in Lead avR. This rare ECG appearance occurs in about 2.0% of patients with acute extensive anterior myocardial infarction due to proximal LAD occlusion. It carries a high positive predictive value (95.2–100%) for acute coronary occlusion.^3^ Currently, de Winter syndrome is not classified under ST-elevation ACS but should be managed similarly to STEMI due to its equal risk profile.

Wellens syndrome, first described by Wellens and co-workers^2^ in 1982,is characterized by distinct electrocardiographic T-wave changes primarily in the pre-cordial leads (predominantly V2–V4, but extending potentially to V1–V6). These changes indicate critical proximal stenosis in LAD. If left untreated, it can progress to a large anterior myocardial infarction. There are two types of T-wave changes observed: symmetric, bidirectional T waves (Type A) and deeply inverted T waves (Type B).^4^ Wellens syndrome is considered an acute equivalent of STEMI and warrants early PCI intervention. Although cardiac ischaemia in other vessels can also present with inverted T waves, in Wellens syndrome, the amplitude of the inversion usually is more negative. The Type B pattern is more common than Type A and may occur more frequently in women.^5^ In this case, Wellens ECG changes occurred after PCI, the patient chest pain relieved, and the ECG changes may be explained by reperforation injury, myocardial stunning, or oedema, other than ischaemia resulting from LAD stenosis.

Both de Winter and Wellens syndromes are recognized as high-risk ECG changes, serving as equivalents to STEMI associated with proximal LAD artery occlusion, necessitating urgent angiography and reperfusion. In Wellens syndrome, T-wave changes typically manifest during the pain-free interval. However, in this case, we observed Wellens ECG changes post-PCI, with few articles documenting the sequential ECG changes of de Winter syndrome following PCI. These ECG alterations can be attributed to reperfusion injury, myocardial stunning, or oedema.^6^ The Wellens ECG pattern arises from alterations in myocardial cell repolarization following severe ischaemia, involving increased opening of adenosine triphosphate-sensitive potassium ion channels and shorter endocardial repolarization compared to epicardial repolarization, resulting in deep T-wave inversion and QT interval prolongation. The Wellens ECG pattern may appear either before or after PCI. When occurring before PCI, T-wave changes in Wellens syndrome typically occur during pain-free intervals, suggesting spontaneous coronary artery revascularization.^7^ In contrast, de Winter syndrome usually manifests during episodes of chest pain, indicating acute severe coronary stenosis. Furthermore, de Winter syndrome is more specific for proximal LAD stenosis or occlusion, while Wellens ECG changes are non-specific. Therefore, for high-risk ACS patients, continuous monitoring of ECG changes is crucial to promptly initiate emergency reperfusion therapy.

## Supplementary Material

ytaf139_Supplementary_Data

## Data Availability

The data underlying this article are available in its online Supplementary material. Additional data could be provided upon reasonable requests.

## Lead author biography

Xianghong Ma, MD, PhD, is a Chief Physician of Cardiology Department of the Second Hospital of Tianjin Medical University. She has rich clinical experience in cardiology, and she is good at interventional treatment of coronary heart disease.

## Supplementary material

**Consent**: The authors confirm that a written consent for the submission and publication of this case report, including images and associated text, has been obtained from the patients in line with the COPE guidelines.

**Funding:** The work was funded by Tianjin Key Medical Discipline (Specialty) Construction Projet (TJYXZDXK-029A).
